# Associations Between Natural Language Processing–Enriched Social Determinants of Health and Suicide Death Among US Veterans

**DOI:** 10.1001/jamanetworkopen.2023.3079

**Published:** 2023-03-15

**Authors:** Avijit Mitra, Richeek Pradhan, Rachel D. Melamed, Kun Chen, David C. Hoaglin, Katherine L. Tucker, Joel I. Reisman, Zhichao Yang, Weisong Liu, Jack Tsai, Hong Yu

**Affiliations:** 1 Manning College of Information and Computer Sciences, University of Massachusetts Amherst; 2 Department of Epidemiology, Biostatistics and Occupational Health, McGill University, Montreal, Quebec, Canada; 3 Department of Biological Sciences, University of Massachusetts Lowell; 4 Department of Statistics, University of Connecticut, Storrs; 5 Center for Population Health, Uconn Health, Farmington, Connecticut; 6 Department of Population and Quantitative Health Sciences, University of Massachusetts Chan Medical School, Worcester; 7 Department of Biomedical & Nutritional Sciences, University of Massachusetts Lowell; 8 Center for Healthcare Organization & Implementation Research, Veterans Affairs Bedford Healthcare System, Bedford, Massachusetts; 9 Miner School of Computer and Information Sciences, University of Massachusetts Lowell; 10 Center for Biomedical and Health Research in Data Sciences, University of Massachusetts Lowell; 11 National Center on Homelessness Among Veterans, US Department of Veterans Affairs, Tampa, Florida; 12 School of Public Health, University of Texas Health Science Center at Houston

## Abstract

**Importance:**

Social determinants of health (SDOHs) are known to be associated with increased risk of suicidal behaviors, but few studies use SDOHs from unstructured electronic health record notes.

**Objective:**

To investigate associations between veterans’ death by suicide and recent SDOHs, identified using structured and unstructured data.

**Design, Setting, and Participants:**

This nested case-control study included veterans who received care under the US Veterans Health Administration from October 1, 2010, to September 30, 2015. A natural language processing (NLP) system was developed to extract SDOHs from unstructured clinical notes. Structured data yielded 6 SDOHs (ie, social or familial problems, employment or financial problems, housing instability, legal problems, violence, and nonspecific psychosocial needs), NLP on unstructured data yielded 8 SDOHs (social isolation, job or financial insecurity, housing instability, legal problems, barriers to care, violence, transition of care, and food insecurity), and combining them yielded 9 SDOHs. Data were analyzed in May 2022.

**Exposures:**

Occurrence of SDOHs over a maximum span of 2 years compared with no occurrence of SDOH.

**Main Outcomes and Measures:**

Cases of suicide death were matched with 4 controls on birth year, cohort entry date, sex, and duration of follow-up. Suicide was ascertained by National Death Index, and patients were followed up for up to 2 years after cohort entry with a study end date of September 30, 2015. Adjusted odds ratios (aORs) and 95% CIs were estimated using conditional logistic regression.

**Results:**

Of 6 122 785 veterans, 8821 committed suicide during 23 725 382 person-years of follow-up (incidence rate 37.18 per 100 000 person-years). These 8821 veterans were matched with 35 284 control participants. The cohort was mostly male (42 540 [96.45%]) and White (34 930 [79.20%]), with 6227 (14.12%) Black veterans. The mean (SD) age was 58.64 (17.41) years. Across the 5 common SDOHs, NLP-extracted SDOH, on average, retained 49.92% of structured SDOHs and covered 80.03% of all SDOH occurrences. SDOHs, obtained by structured data and/or NLP, were significantly associated with increased risk of suicide. The 3 SDOHs with the largest effect sizes were legal problems (aOR, 2.66; 95% CI, 2.46-2.89), violence (aOR, 2.12; 95% CI, 1.98-2.27), and nonspecific psychosocial needs (aOR, 2.07; 95% CI, 1.92-2.23), when obtained by combining structured data and NLP.

**Conclusions and Relevance:**

In this study, NLP-extracted SDOHs, with and without structured SDOHs, were associated with increased risk of suicide among veterans, suggesting the potential utility of NLP in public health studies.

## Key Points

**Question:**

Are social determinants of health (SDOHs), extracted from both structured and unstructured clinical data, associated with an increased risk of suicide death among US veterans?

**Findings:**

In this case-control study of 8821 cases and 35 284 matched controls, SDOHs from both structured data and unstructured data (extracted using a natural language processing system) were associated with an increased risk of suicide death.

**Meaning:**

The findings of this study suggest that SDOHs are risk factors for suicide among the US veterans and that natural language processing can be leveraged to extract SDOH information from unstructured data.

## Teaser

This case-control study assesses suicide risk among veterans by social determinants of health (SDOHs) identified using both structured data and unstructured data in the electronic health record extracted using natural language processing.

## Introduction

Suicide is among the leading causes of death among US residents, accounting for 47 511 deaths in 2019 alone.^1^ Nationwide, deaths by suicide increased 30% from 1999 to 2016.^2^ In 2013 alone, the total cost of suicides and suicide attempts in the United States was estimated to be $93.5 billion.^3^ In the past decade, suicide rates have been consistently higher among veterans than nonveterans, and even more alarming, the suicide rate among veterans has risen faster than among nonveteran adults.^4^

Social determinants of health (SDOHs), which include conditions such as socioeconomic status, access to healthy food, education, housing, and physical environment,^5^ are strong predictors of suicidal behaviors (ideation, attempt, and death).^6,7,8,9^ For example, social disruptions (eg, relationship dissolution, financial insecurity, legal problems, or exposure to childhood adversity) are well-known to instigate suicidal behavior.^6,10,11,12^ To formulate policies addressing suicide prevention, one must go beyond identifying predictors by determining the magnitude of the association of SDOHs with suicide. A key impediment to this has been the lack of comprehensive and reliably available SDOH information in large population-based databases, where investigators have traditionally relied on structured data. Structured data often lack completeness regarding SDOH information, specifically, when they are designed for billing purposes. A recent study showed that unstructured data contain about 90 times more SDOH information than structured data.^13^

Although existing studies identified a range of common risk-factors for suicide using structured data from electronic health records (EHRs),^14,15,16,17,18^ unstructured EHR data received little attention in investigating potential associations between suicide and SDOH. Therefore, in a nested case-control study, we used both structured data (*International Classification of Diseases *[*ICD*] codes, stop codes) and unstructured data (clinical notes, processed by a novel natural language processing [NLP] system) from the large EHR system of the US Veterans Health Administration (VHA) to examine the association of 9 SDOH factors with risk of suicide.

## Methods

### Data Source

This study used the EHR database from the VHA Corporate Data Warehouse (CDW). With a primary obligation to provide medical services to all eligible US veterans, the VHA is the largest integrated health care network in the country; its EHR system spans more than 1200 health care facilities, including medical centers and clinics.^19^ The VHA database includes patient demographic information, medication, diagnoses, procedures, clinical notes, and billing. Our study protocol was approved by the institutional review board of US Veterans Affairs (VA) Bedford Health Care, and we obtained a waiver of informed consent due to minimal risk to participants. The Strengthening the Reporting of Observational Studies in Epidemiology (STROBE)^20^ reporting guidelines were followed.

### Study Population

As with any state-of-the-art NLP system, analyzing all patients in our base cohort presented a computational challenge. In addition, we studied multiple exposures. Therefore, we used a nested case-control design and risk-set sampling to match the controls to the cases. This approach facilitates studying associations of exposures (eg, SDOHs) with rare events such as suicide outcome.^21^

The base cohort consisted of all veterans for whom the VHA database had any record of service during the period between October 1, 2010 (start of fiscal year [FY] 2011), and September 30, 2015 (end of FY2015). Each patient’s cohort entry date was defined as the latest of these dates: when the patient had 2 years of medical history in the database, the patient’s 18th birthday, or the start of FY2011. The end of follow-up was defined as the earliest of the following: suicide, death from other causes, end of last record for the patient, or end of the study period (end of FY2015). We excluded all patients who had prior suicide attempts^22^ or no EHR notes before their cohort entry dates. Patients with missing or erroneous demographic information and those older than 100 years were also excluded.

Cases consisted of all patients in the base cohort who died by suicide (according to National Death Index^23^ with *International Statistical Classification of Diseases and Related Health Problems, Tenth Revision *[*ICD-10*] codes X60-X84, Y87.0, and/or U03 as underlying cause of death) during FY2011 to FY2015. Each case was randomly matched, with replacement, to 4 control participants from those who were still alive. The matching criteria were (1) birth year (±3 years), (2) cohort entry FY, (3) sex, and (4) duration of follow-up (same or longer than the case participant). By design, a case participant could serve as a control participant for another case participant who committed suicide at an earlier date, and a patient could be a control participant for multiple case participants. The index date for each case was defined as the date of suicide, and each control was assigned the same index date as their corresponding case. All data analyses were performed in May 2022.

### NLP

A unique aspect of this study is the integration of an NLP system to extract SDOH, behavioral, and other relevant factors from EHR notes. We implemented a multitask learning (MTL) framework based on the pretrained language model: RoBERTa.^24^ RoBERTa is an improved version of bidirectional encoder representations from transformers (BERT)^25^ which has been shown to outperform all other NLP systems across a wide range of downstream tasks. To train our MTL model, we collected 4646 EHR notes from 1393 patients (excludes all patients from our base cohort) who received treatment at the VHA and died between FY2009 and FY2017. Under expert supervision, 3 trained annotators annotated these notes for 13 distinct SDOH, behavioral, and relevant factors. This is in accordance with the recent clinical practice guideline issued jointly by the VA and the Department of Defense.^26^ Our MTL model was fine-tuned on this data set for 3 joint tasks: factor, presence, and period identification. eAppendix 1 in Supplement 1 provides a detailed description of our note selection, annotation process, and MTL model performance.

We used this fine-tuned MTL model to extract 13 factors for our study population, 8 of which were SDOHs (eAppendix 1 in Supplement 1). For each patient visit, we used 7 types of notes: emergency department notes, nursing assessments, primary care notes, hospital admission notes, inpatient progress notes, pain management, and discharge summaries. For multiple notes within an observation window (covariate or exposure assessment periods), we merged multiple extractions of each factor and prioritized presence yes over presence no. Each model-extracted factor was dichotomized using the following strategy:

1. An extracted factor with presence yes and period current was coded as 1.
2. All other extractions of the same factor were coded as 0, including factors with missing presence and period attributes.
3. Notes with no extracted factors and patients with no notes were coded as 0.

### SDOH Extraction

We extracted SDOHs from both unstructured EHR text (using NLP) and structured data (using *ICD-10* codes and VHA stop codes (eAppendix 2 in Supplement 1). The NLP-extracted SDOHs comprised 8 factors: social isolation, job or financial insecurity, housing instability, legal problems, violence, barriers to care, transition of care, and food insecurity; the structured SDOH comprised 6 factors:^6^ social or familial problems, employment or financial problems, housing instability, legal problems, violence, and nonspecific psychosocial needs. We combined these 2 groups to have 9 distinct factors, 5 of which were represented in both sets, whereas barriers to care, transition of care, and food insecurity were found only in the NLP-extracted SDOH and nonspecific psychosocial needs was found only in the structured SDOH.

### Exposure and Covariate Assessment

To focus on the association of recent SDOH events with suicide, we considered a patient’s exposures to the aforementioned 9 SDOHs in the 2 years before the index date but not prior to the cohort entry date. The covariate assessment period was 2 years prior to the cohort entry date. Potential covariates were sociodemographic variables, clinical comorbidities, and mental health disorders. Sociodemographic variables included race (American Indian or Alaska Native, Asian, Black, Native Hawaiian or other Pacific Islander, White, or unknown), age, and marital status. Race was self-reported, and we included it in compliance with prior studies related to suicidal behaviors.^6,8,11,14^ From the Charlson Comorbidity Index,^27^ we included 17 clinical comorbidities: acute myocardial infarction, congestive heart failure, peripheral vascular disease, cerebrovascular disease, dementia, chronic obstructive pulmonary disease, rheumatoid disease, peptic ulcer disease, mild liver disease, diabetes without complications, diabetes with complications, hemiplegia or paraplegia, kidney disease, cancer, moderate or severe liver disease, metastatic solid tumor, and AIDS/HIV. We considered 7 mental health disorders: major depressive disorder, alcohol use disorder, drug use disorder, anxiety disorder, posttraumatic stress disorder, schizophrenia, and bipolar disorder.^6^ We also added psychiatric symptoms, substance abuse, pain, and patient disability that were extracted by our NLP system. Additionally, for each model with a specific SDOH as the exposure, the list of covariates included all SDOHs in its group (ie, NLP-extracted, structured, or combined).

### Statistical Analysis

We calculated the crude incidence rate of suicide along with 95% CIs based on the Poisson distribution. For each SDOH exposure variable, we fit a conditional logistic regression model with death by suicide as the outcome. In addition to matching for birth year, cohort entry date, sex, and duration of follow-up, we adjusted all models for the specified covariates (potential confounders). This procedure yielded a total of 23 models: 8 for NLP-extracted SDOHs, 6 for structured SDOHs, and 9 when combined. Following the same process, we also considered exposure to 2 SDOHs at the same time, yielding a total of 79 models: 28 for NLP-extracted SDOHs, 15 for structured SDOHs, and 36 when combined. The variance inflation factor (VIF)^28^ showed no evidence of collinearity (no VIF exceeded 3) among the covariates for any model. We report adjusted odds ratios (aORs) with 95% CIs for each exposure. All analyses used RStudio version 0.99.902 with R version 3.6.0 (R Project for Statistical Computing).

## Results

Our base cohort consisted of 6 122 785 Veterans (Figure, A) from all VA health care facilities in the United States or its territories; the majority were white (76.99%) and male (92.23%). Veterans 50 years of age or older comprised 75.78% of the population. We had a mean (SD) follow-up of 3.87 (1.39) years, generating 23 725 382 person-years, with 8821 deaths by suicide (crude incidence rate 37.18 [95% CI, 36.41-37.96] per 100 000 person-years). eAppendix 3 in Supplement 1 reports detailed characteristics of the base cohort. This case-control cohort consisted of 8821 case participants and 35 284 matched control participants (34 404 unique individuals, of whom 846 served as control participants for more than 1 case participant) from 43 188 veterans who were served at 908 VA facilities. In the case-control cohort, most participants were also White (34 930 [79.20%], with 6227 [14.12%] Black veterans) and male (42 540 [96.45%]) with a mean (SD) age of 58.64 (17.41) years. Compared with case participants, control participants had a higher percentage of Black individuals (5742 [16.27%] vs 485 [5.50%]) and lower percentage of White individuals (27 125 [76.88%] vs 7805 [88.48%]). Detailed characteristics are shown in Table 1.

**Figure.**
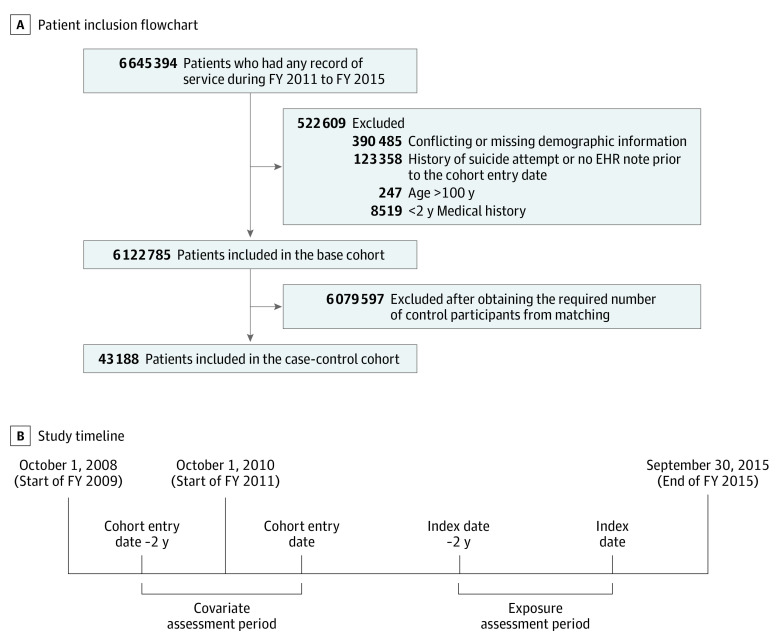
Construction of the Cohort and Study Timeline EHR indicates electronic health record; FY, fiscal year.

**Table 1.** Summary Statistics for Case and Control Participants

Characteristic	Participants, No. (%)	
	Case (n = 8821)	Control (n = 35 284)^a^
Race		
American Indian or Alaska Native	68 (0.77)	240 (0.68)
Asian	42 (0.48)	322 (0.91)
Black	485 (5.50)	5742 (16.27)
Native Hawaiian or other Pacific Islander	70 (0.79)	345 (0.98)
White	7805 (88.48)	27 125 (76.88)
Unknown	351 (3.98)	1510 (4.28)
Sex		
Male	8508 (96.45)	34 032 (96.45)
Female	313 (3.55)	1252 (3.55)
Age, y		
18-29	774 (8.77)	3085 (8.74)
30-39	714 (8.09)	2842 (8.05)
40-49	1000 (11.34)	3911 (11.08)
50-59	1587 (17.99)	6163 (17.47)
60-69	2278 (25.82)	9597 (27.20)
70-79	1347 (15.27)	5271 (14.94)
80-100	1121 (12.71)	4415 (12.51)
Marital status		
Married	2539 (28.78)	8846 (25.07)
Single	1028 (11.65)	2002 (5.67)
Divorced	1871 (21.21)	3117 (8.83)
Widowed	466 (5.28)	991 (2.81)
Unknown	2917 (33.06)	20 328 (57.61)
Comorbidities from Charlson Comorbidity Index		
Acute myocardial infarction	58 (0.66)	207 (0.59)
Congestive heart failure	213 (2.41)	812 (2.30)
Peripheral vascular disease	281 (3.19)	948 (2.69)
Cerebrovascular disease	275 (3.12)	1081 (3.06)
Dementia	17 (0.19)	153 (0.43)
COPD	962 (10.91)	2859 (8.10)
Rheumatoid disease	57 (0.65)	249 (0.71)
Peptic ulcer disease	47 (0.53)	127 (0.36)
Mild liver disease	188 (2.13)	496 (1.41)
Diabetes without complications	1108 (12.56)	5408 (15.33)
Diabetes with complications	257 (2.91)	1246 (3.53)
Hemiplegia or paraplegia	59 (0.67)	186 (0.53)
Kidney disease	131 (1.49)	682 (1.93)
Cancer (any malignant neoplasm)	489 (5.54)	1673 (4.74)
Moderate or severe liver disease	15 (0.17)	47 (0.13)
Metastatic solid tumor	28 (0.32)	74 (0.21)
AIDS/HIV	26 (0.29)	141 (0.40)
Mental health comorbidities		
Major depressive disorder	2191 (24.84)	4790 (13.58)
Alcohol use disorder	778 (8.82)	1470 (4.17)
Drug use disorder	425 (4.82)	924 (2.62)
Anxiety disorder	880 (9.98)	1953 (5.54)
PTSD	1208 (13.69)	3676 (10.42)
Schizophrenia	262 (2.97)	525 (1.49)
Bipolar disorder	708 (8.03)	1101 (3.12)
NLP-extracted non-SDOH factors		
Patient disability	3718 (42.15)	13 643 (38.67)
Substance abuse	5408 (61.31)	20 539 (58.21)
Psychiatric symptoms	5436 (61.63)	19 954 (56.55)
Pain	5664 (64.21)	22 048 (62.49)

Abbreviations: COPD, chronic obstructive pulmonary disease; NLP, natural language processing; PTSD, posttraumatic stress disorder; SDOH, social determinant of health.

^a^ Number of control participants, not number of unique patients.

The structured SDOHs had low prevalence compared with their NLP-extracted counterparts (Table 2). For example, of 12 691 veterans exposed to social problems, only 4101 (32.31%) were identified by structured SDOHs whereas NLP identified 10 889 (85.80%). We found similar results for the remaining combined SDOHs (eAppendix 4 in Supplement 1). As covariates, across all the 5 common SDOHs, considering combined SDOHs as the gold standard, NLP-extracted SDOH had a mean (SD) coverage of 78.86% (10.89%) compared with 36.02% (9.05%) from structured SDOH. As exposures, the numbers were 80.03% (9.68%) and 38.17% (8.57%) respectively. All SDOHs occurred more frequently among case participants than among control participants. Moreover, for most SDOHs, we found more occurrences during the exposure assessment period than during the covariate assessment period.

**Table 2.** Summary Statistics of SDOH Factors as Covariate and as Exposure

SDOH	Participants, No. (%)											
	As covariate						As exposure					
	NLP-extracted		Structured data		Combined		NLP-extracted		Structured data		Combined	
	Case	Control	Case	Control	Case	Control	Case	Control	Case	Control	Case	Control
Social problems^a^	2584 (29.29)	7891 (22.36)	833 (9.44)	2691 (7.63)	2938 (33.31)	9227 (26.15)	3080 (34.92)	7809 (22.13)	1291 (14.64)	2810 (7.96)	3517 (39.87)	9174 (26.00)
Financial problems^b^	2016 (22.85)	6052 (17.15)	603 (6.84)	1661 (4.71)	2206 (25.01)	6652 (18.85)	2274 (25.78)	5710 (16.18)	882 (10.00)	1858 (5.27)	2513 (28.49)	6381 (18.08)
Housing instability	1399 (15.86)	4266 (12.09)	592 (6.171)	1657 (4.70)	1638 (18.57)	5009 (14.20)	2005 (22.73)	5027 (14.25)	953 (10.80)	2030 (5.75)	2314 (26.23)	5852 (16.59)
Legal problems	730 (8.28)	1644 (4.66)	530 (6.01)	1390 (3.94)	1072 (12.15)	2695 (7.64)	1025 (11.62)	1760 (4.99)	886 (10.04)	1520 (4.31)	1567 (17.76)	2831 (8.02)
Violence	1141 (12.94)	3228 (9.15)	604 (6.85)	1981 (5.61)	1560 (17.69)	4784 (13.56)	1700 (19.27)	3519 (9.97)	800 (9.07)	1734 (4.91)	2108 (23.90)	4839 (13.71)
Barriers to care	1465 (16.61)	4311 (12.22)	NA	NA	1465 (16.61)	4311 (12.22)	2041 (23.14)	5083 (14.41)	NA	NA	2041 (23.14)	5083 (14.41)
Transitions of care	4838 (54.85)	17 965 (50.92)	NA	NA	4838 (54.85)	17 965 (50.92)	5181 (58,73)	18 175 (51.51)	NA	NA	5181 (58,73)	18 175 (51.51)
Food insecurity	291 (3.30)	910 (2.58)	NA	NA	291 (3.30)	910 (2.58)	411 (4.66)	942 (2.67)	NA	NA	411 (4.66)	942 (2.67)
Nonspecific psychosocial needs	NA	NA	1198 (13.58)	3808 (10.79)	1198 (13.58)	3808 (10.79)	NA	NA	1670 (18.93)	3749 (10.63)	1670 (18.93)	3749 (10.63)

Abbreviations: NA, not applicable; NLP, natural language processing; SDOH, social determinants of health.

^a^ Social problems indicates social or familial problems from structured data with social isolation from NLP-extracted data.

^b^ Financial problems indicates employment or financial problems from structured data with job or financial insecurity from NLP-extracted data.

All 8 NLP-extracted SDOH were significantly associated with increased risk of death by suicide (Table 3). Legal problems had the largest estimated effect size (more than twice the risk of those with no exposure; aOR, 2.62; 95% CI. 2.38-2.89), followed by violence (aOR, 2.34; 95% CI, 2.17-2.52) and social isolation (aOR, 1.94; 95% CI, 1.83-2.06). All 7 structured SDOHs also showed significant associations; again, legal problems had the highest aOR (2.63; 95% CI, 2.37-2.91). Similarly, all combined SDOHs showed strong associations, and the top 3 risk factors were legal problems (aOR, 2.66; 95% CI, 2.46-2.89), violence (aOR, 2.12; 95% CI, 1.98-2.27), and nonspecific psychosocial needs (aOR, 2.07; 95% CI, 1.92-2.23).

**Table 3.** Associations of SDOH With Veterans’ Death by Suicide

SDOH factors	aOR (95% CI)^a^		
	NLP-extracted	Structured	Combined
Social problems^b^	1.94 (1.83-2.06)	2.11 (1.94-2.29)	1.95 (1.84-2.07)
Financial problems^c^	1.91 (1.79-2.04)	2.18 (1.97-2.42)	1.92 (1.80-2.05)
Housing instability	1.90 (1.78-2.03)	2.28 (2.06-2.53)	1.93 (1.80-2.06)
Legal problems	2.62 (2.38-2.89)	2.63 (2.37-2.91)	2.66 (2.46-2.89)
Violence	2.34 (2.17-2.52)	1.96 (1.77-2.16)	2.12 (1.98-2.27)
Barriers to care	1.86 (1.74-1.99)	NA	1.86 (1.74-1.98)
Transition of care	1.53 (1.44-1.62)	NA	1.51 (1.43-1.60)
Food insecurity	1.85 (1.62-2.11)	NA	1.85 (1.62-2.11)
Nonspecific psychosocial needs	NA	2.09 (1.94-2.25)	2.07 (1.92-2.23)

Abbreviations: aOR, adjusted odds ratio; NA, not applicable; NLP, natural language processing; SDOH, social determinants of health.

^a^ Each model was adjusted for sociodemographic variables, psychiatric symptoms, substance abuse, pain, patient disability, clinical comorbidities, and all SDOH in its group.

^b^ Social problems indicates social or familial problems from structured data with social isolation from NLP-extracted data.

^c^ Financial problems indicates employment or financial problems from structured data with job or financial insecurity from NLP-extracted data.

When considering simultaneous exposure to 2 SDOHs, we found all combinations of SDOHs to be strongly associated with increased risk of death by suicide (eAppendix 5 in Supplement 1), regardless of the SDOH extraction process. For NLP-extracted SDOHs, the highest aOR was for exposure to legal problems and violence (aOR, 3.44; 95% CI, 3.03-3.89). For structured SDOHs, exposure to financial problems and violence had the highest aOR (3.54; 95% CI, 2.87-4.36). Combined SDOHs also showed similar associations.

## Discussion

To our knowledge, this is the first large-scale study that used both structured and unstructured EHR data to investigate the association between veterans’ suicide and SDOHs. We developed and deployed an NLP system to extract SDOH from unstructured clinical notes and found that all NLP-extracted SDOHs were strongly associated with increased odds of suicide. We observed similar results for structured and combined SDOH.

Although many studies have explored the consequences of various SDOHs over different clinical outcomes,^14,29,30,31^ very few have examined the association of SDOHs with increased risk of suicide, or the magnitude of such associations, if any. In a nested case-control study of veterans, Kim et al^8^ used medical record review to examine SDOHs. However, their study focused on a high-risk population of those with depression and had a small sample size (636 participants). In contrast, in a large cross-sectional study of veterans, Blosnich et al^6^ found a dose-response–like association with SDOHs for both suicidal ideation and attempt. However, cross-sectional studies are unsuitable for investigating rare events such as suicide.^32^ Most importantly, neither of these studies used the rich information provided by clinical notes. On the other hand, in a case-control study, Dobscha et al^33^ extracted SDOHs from clinical notes through manual record review and found no evidence of association between veteran suicide and SDOHs. They had a relatively small sample size (783 participants) and included only male patients.

An important contribution of our study is the development of an NLP system to extract SDOHs from unstructured EHR text. Our NLP system extracted a considerable number of SDOHs that were not available from the structured data fields (eAppendix 4 in Supplement 1). These can help clinicians identify crucial SDOH information that they would otherwise miss. However, NLP-extracted SDOHs did not cover all structured SDOHs. Across the 5 common SDOHs, NLP extracted 44.91% of the structured SDOH information as covariates whereas as exposures it extracted 49.92%. This may be due to missing SDOH information in EHR notes or false negatives from the NLP system. Structured data, on the other hand, identified 18.86% of the NLP-extracted SDOH as covariates and 22.85% as exposures. Therefore, taking their unique contributions into account, we suggest combining both structured SDOHs and NLP-extracted SDOHs for assessment.

For the 5 common SDOHs, structured SDOHs consistently showed higher aORs for suicide than NLP-extracted SDOHs. One possible explanation for this might be that in control participants, who are less likely to be sick, clinicians may not be inclined to note their SDOH information in the structured data fields. We hypothesize that clinicians only do so when it is pertaining to the patient’s primary diagnosis or ongoing clinical care, possibly representing a population that has relatively more severe illness than all the patients with identifiable SDOHs in their clinical notes. For example, 14.64% of the case population were exposed to social problems, as identified by the structured data, compared with 34.92% by the NLP system, a 2.4-fold increase (Table 2). However, this goes up to 2.8-fold for control participants (7.96% vs 22.13%). Thus, using NLP-derived SDOH information might reduce information bias, an important problem in assessing psychosocial research questions.

To estimate whether intervening on SDOHs has the potential to change suicide risk, it is necessary to separate its influence from other related factors. In effect, we aimed at emulating the results of an experimental setting where people who experience certain SDOH issues would be enrolled in a trial that randomly assigns whether one receives an intervention. Because such a trial is not available, we relied on observational health data to inform our understanding of suicide. We used epidemiologic methods to adjust for the differences between people exposed to SDOHs and those who were not. We carefully considered several possible confounding health and demographic factors in our design to obtain the best possible estimate of the associations of SDOHs with suicide.

Our work found a strong association of SDOHs with veterans’ risk of suicide using a nested case-control design, in which both the covariate and exposure assessment periods are limited to 2 years. This setup reduces the burden of data processing and NLP extraction and yet provides a valid assessment of the potential associations between (recent) SDOHs and suicide. On the other hand, using longer covariate and exposure assessment periods could provide more information and insights on both short-term (acute) and long-term (persistent) associations of SDOH with suicide. A related problem is that SDOHs change over time; as such, it is more appropriate to treat them as time-varying exposures for longer exposure assessment periods. These time-varying aspects of the problem will be carefully explored in our future work.

### Limitations

Our study has some limitations. First, the VA population does not represent the general US population. However, many studies and innovations from the VHA have been shown to assist non-VHA facilities in adopting better clinical practices.^34,35,36^ Second, there is potential for residual confounding. Third, EHR data might have incomplete or missing SDOH information,^37^ making it challenging to assess the influence of SDOHs on any target outcome. However, most SDOHs with a direct relation to provided care are recorded, so our approach is unlikely to miss important SDOHs when both structured and unstructured data are used.

## Conclusions

To our knowledge, this is the first large-scale study to implement and use an NLP system to extract SDOH information from unstructured EHR data. We found that SDOH are associated with veterans’ risk of death by suicide. Our results also indicate that integrating NLP-based SDOHs can benefit similar analyses by identifying more patients at risk. We strongly believe that analyzing all available SDOH information, including those contained in clinical notes, can help develop a better system for risk assessment and suicide prevention. However, more studies are required to investigate ways of seamlessly incorporating SDOHs into existing health care systems.

## Notes

Supplement 1. **eAppendix 1.** NLP Model Development **eAppendix 2.** *ICD* and Stop Codes for Structured SDOH and Mental Health Disorders **eAppendix 3.** Base Cohort Statistics **eAppendix 4.** SDOH Prevalence **eAppendix 5.** Associations for Concurrent SDOH
eReferences.

Supplement 2.
Data Sharing Statement
