# Supplementary material for: Associations Between Natural Language Processing–Enriched Social Determinants of Health and Suicide Death Among US Veterans
Source: JAMA Netw Open. 2023 Mar 15;6(3):e233079. doi: 10.1001/jamanetworkopen.2023.3079 (PMC10018322; doi:10.1001/jamanetworkopen.2023.3079)
Supplement: Supplement 1. — eAppendix 1. NLP Model Development eAppendix 2. ICD and Stop Codes for Structured SDOH and Mental Health Disorders eAppendix 3. Base Cohort Statistics eAppendix 4. SDOH Prevalence eAppendix 5. Associations for Concurrent SDOH eReferences. [file jamanetwopen-e233079-s001.pdf]

Supplemental Online Content

Mitra A, Pradhan R, Melamed RD, et al. Associations between natural language processing–enriched social determinants of health and suicide death among US veterans. *JAMA Netw Open.* 2023;6(3):e233079. doi:10.1001/jamanetworkopen.2023.3079

eAppendix 1. NLP Model Development

eAppendix 2. *ICD* and Stop Codes for Structured SDOH and Mental Health Disorders

eAppendix 3. Base Cohort Statistics

eAppendix 4. SDOH Prevalence

eAppendix 5. Associations for Concurrent SDOH

eReferences.

This supplemental material has been provided by the authors to give readers additional information about their work.

© 2023 Mitra A et al. *JAMA Network Open.*

Appendix 1. NLP Model Development

Dataset

We used a stratified random sampling approach to sample 3,000 Veterans who received treatment at the VHA and died between fiscal year 2009-2017. Patients were stratified and sampled by several key sociodemographic variables (race, gender, and age), geographic location (Northeast, Midwest, South, and West, 1:1:1:1 ratio), and death by suicide (2:1 ratio to those who did not die from suicide). We also oversampled underrepresented groups such as women and ethnic minorities. For the ease of manual annotation, we further reduced the number of Veterans by sampling 1,393 veterans while maintaining a Veterans with suicide attempt to no attempt ratio of 1:4. For each Veteran, we chose three types of notes – social worker notes, mental health notes and emergency room visit notes.

At first, we conducted a pre-screening phase using a predefined set of keywords to select the most relevant sentences in a note and turned each of them into a three-sentence paragraph by using the previous and next sentences. All these paragraphs came from 4,646 unique EHR notes. Next, these paragraphs were annotated for 13 distinct SDOH, behavioral and other relevant factors by three expert annotators under expert supervision (Table 1). These 13 factors were selected based on expert opinions and the recent clinical practice guideline issued by the Veterans Affairs and Department of Defense [1]. Each factor was further annotated for two attributes, 'presence' (yes, not yes) and 'period' (current, not current).

NLP Extracted Variables	Brief descriptions	Example tokens
Social isolation*	Social and behavioral status to detect loneliness, lack of social and/or family support; marital/relationship status.	Alone, lonely, divorce, widow etc.
Transition of care*	Change of admission status (discharge, transfer etc.); change in medication and/or	Discharge, admission, change in medication, transfer etc.

© 2023 Mitra A et al. *JAMA Network Open.*

	provider.	
Barriers to care*	Transportation issues; communication problems; lack of trust or rapport; intellectual disability	Transportation issues, garbled speech, communication problems etc.
Financial insecurity*	Financial issues; job problems; poverty	Unemployed, poor, unemployment, rehabilitation etc.
Housing instability*	Housing issues	Eviction, homeless, homelessness etc.
Food insecurity*	Poor diet and/or nutrition; lack of access to proper meal; dependency on food charities/voucher/stamps	Hungry, pantry, starvation, food voucher etc.
Violence*	Availability and/or access to lethal means; bullying; domestic violence; any harassment/abuse/trauma; racism; homicidal ideation; feeling scared/unsafe	Firearms, violence, assault, weapon, abuse, homicidal, racism etc.
Legal problems*	Imprisonment; court-related matters; detentions; disciplinary action; restraining orders; brushes with the law; criminal charges; any violation of law	Imprisonment, parole, arrested, felony, investigation, prison etc.
Substance abuse	Drug use disorder; alcohol consumption; alcohol use disorder; addiction; overdose	Alcohol, tobacco, heroin, cocaine, smoking, overdose etc.
Psychiatric symptoms	Hopelessness; Insomnia; Problem solving difficulty; decreased psychosocial	PTSD, depression, anxiety, schizophrenia, insomnia, hallucination etc.

© 2023 Mitra A et al. *JAMA Network Open.*

	functioning; psychiatric hospitalization; eating disorder; mention of any psychiatric disease	
Pain	Physical pain	Pain, suffering, hurting, discomfort etc.
Patient disability	Reliance on assistive devices and/or disability pay; service-connected ratings	Disabled, blind, hearing loss, wheelchair etc.
Suicide outcome	Suicide attempt and/or ideation	Feel like shooting myself, no desire to live, better off dead

*SDOH

eTable 1. Examples of NLP-extracted factors.

Task and Experimental Setup

We designed this as a sequence labeling problem where, given a sentence from a note, our goal was to label each word with the appropriate SDOH/behavioral factor and detect the two attributes – presence and period. To be specific, we implemented a multi-task learning framework. A 60:20:20 split was used for the train, validation, and test sets. We experimented with four pretrained language models, namely, RoBERTa [2], BioBERT [3], Bio+Clinical BERT [4] and EhrBERT [5], and for each, we added three heads on top, to identify factors and their attributes jointly. Our code is publicly available at https://github.com/avipartho/SequenceLabelingWithMultiTaskLEarning.

Result

We evaluated the model performance using precision, recall and F-score. We considered both exact matching (i.e., both the span boundary and entity type match with that of the ground truth) and relaxed matching (i.e., the span boundaries overlap and entity types match) to assess the model performance. In our experiments, RoBERTa achieved the best performance among all the models. The results for RoBERTa are shown in eTable 2, eTable 3 and eTable 4.

© 2023 Mitra A et al. *JAMA Network Open.*

SDOH/behavioral Factors	Exact			Relaxed		
	Precision	Recall	F-score	Precision	Recall	F-score
Social isolation*	85.45	88.04	86.73	92.05	94.84	93.42
Transition of care*	80.07	91.92	85.59	81.21	93.23	86.81
Barriers to care*	54.66	62.93	58.50	68.43	78.78	73.24
Financial insecurity*	64.52	76.04	69.81	74.43	87.71	80.53
Housing instability*	74.37	82.24	78.11	81.06	89.64	85.13
Food insecurity*	66.87	76.43	71.33	70.63	80.71	75.33
Violence*	67.01	74.71	70.65	79.71	88.88	84.04
Legal problems*	64.45	68.86	66.58	82.35	87.98	85.07
Substance abuse	70.75	78.61	74.48	82.32	91.47	86.65
Psychiatric symptoms	74.72	82.64	78.48	82.46	91.20	86.61
Pain	77.82	85.88	81.65	86.86	95.86	91.14
Patient disability	85.91	89.35	87.60	90.41	94.03	92.19
Suicide outcome	67.43	73.89	70.51	81.12	88.89	84.82
Micro	75.03	82.36	78.52	83.14	91.27	87.02
Macro	71.85	79.35	75.39	81.00	89.48	85.00

eTable 2. Performance of our MTL model – factor identification.

Presence	Exact			Relaxed		
	Precision	Recall	F-score	Precision	Recall	F-score
Yes	71.86	77.05	74.36	81.20	87.06	84.02
Not yes	74.14	76.43	75.27	79.76	82.22	80.97
Micro	72.68	76.82	74.69	80.68	85.28	82.92
Macro	73.00	76.74	74.82	80.48	84.64	82.50

© 2023 Mitra A et al. *JAMA Network Open.*

eTable 3. Performance of our MTL model – presence identification.

Presence	Exact			Relaxed		
	Precision	Recall	F-score	Precision	Recall	F-score
Current	75.65	79.84	77.69	83.78	88.42	86.04
Not current	58.93	64.94	61.79	68.28	75.24	71.59
Micro	73.69	78.16	75.86	81.96	86.94	84.38
Macro	67.29	72.39	69.74	76.03	81.83	78.81

eTable 4. Performance of our MTL model – period identification.

Appendix 2. *ICD* and Stop Codes for structured SDOH and Mental Health Disorders

SDOH

SDOH factors	ICD-9 Codes	Stop Codes
Social or familial problems	V60.6, V60.89, V62.3, V62.89, V60.3, V61, V62.4	-
Employment or financial problems	V60.2, V60.89, V60.9, V62.0, V62.1, V62.29	208, 222, 535, 555, 568, 574
Housing instability	V60.0-2, V60.89	504, 507, 508, 511, 522, 528, 529, 530, 555, 556, 590
Legal problems	V62.5, V62.89, E849.7	591, 592
Violence	E904.0, E960.0-1, E961-E977, E979, E990.0-3, E990.9, E991.0-9, E992.0-3, E992.8-9, E993.0-9, E994.0-3, E994.8-9, E995.0-4, E995.8-9, E996.0-3, E996.8-9, E997.0-3, E997.8-9, E998.0-1, E998.8-9, E999.0-1, V15.41-42, V15.49, V71.5, V71.81, 995.50-54, 995.80-85	524
Non-specific psychosocial needs	V62.29, V62.3-6, V62.81, V62.89, V62.9	-

eTable 5. ICD and stop codes for structured SDOH.

Comorbidities

Mental Health Disorders	ICD-9 Codes
Major Depressive disorder	293.83, 296.2, 296.3, 296.9, 298.0, 300.4, 301.12, 309.0,

© 2023 Mitra A et al. *JAMA Network Open.*

	309.1, and 311
Alcohol Use Disorder	291.0-5, 291.8-9, 303.0-303.9, 305.0, 357.5, 425.5, 571.0-3, 535.3, V11.3
Drug Use Disorder	292.0-1, 304.0-304.9, 305.2-305.8
Anxiety Disorder	300.0, 300.1, 300.2, 799.2
Posttraumatic Stress Disorder	309.81
Schizophrenia	295.0-295.9, V11.0
Bipolar disorder	296.0, 296.1, 296.4-7, 296.80, 296.81, 296.82, 296.89, 296.90, 296.99, V11.1

eTable 6. ICD codes for mental health disorders

© 2023 Mitra A et al. *JAMA Network Open.*

eAppendix 3. Base Cohort Statistics

	**Base Cohort (N=6,122,785)**	**%**
Race		
White	4,713,683	76.99%
Black	997,035	16.28%
Asian	58,075	0.95%
Native Hawaiian or Other Pacific Islander	50,210	0.82%
American Indian	44,028	0.72%
Unknown	259,754	4.24%
Gender		
Male	5,646,838	92.23%
Female	475,947	7.77%
Age		
18-29	403,618	6.59%
30-39	435,592	7.11%
40-49	640,441	10.46%
50-59	1,072,726	17.52%

60-69	1,896,202	30.97%
70-79	938,691	15.33%
80-100	735,515	12.01%
Marital Status		
Married	1,672,089	27.31%
Single	330,837	5.40%
Divorced	598,341	9.77%
Widowed	200,842	3.28%
Unknown	3,320,676	54.23

eTable 7. Summary Statistics of the Base Cohort

eAppendix 4. SDOH Prevalence

SDOH	NLP only	Structured data only	Present in both
Social problems	71.03%	13.89%	15.08%
Financial insecurity	74.44%	8.92%	16.64%
Housing insecurity	66.17%	14.77%	19.06%
Legal problems	49.03%	36.98%	13.99%
Violence	59.25%	31.13%	9.62%

eTable 8. Prevalence of Combined SDOH Factors by Source (as Covariates)

SDOH	NLP only	Structured data only	Present in both
Social problems	67.69%	14.20%	18.11%
Financial insecurity	69.19%	10.23%	20.58%
Housing insecurity	63.47%	13.89%	22.64%
Legal problems	45.29%	36.68%	18.03%
Violence	63.52%	24.87%	11.61%

eTable 9. Prevalence of Combined SDOH Factors by Source (as Exposures)

© 2023 Mitra A et al. *JAMA Network Open.*

eAppendix 5. Associations for Concurrent SDOH

SDOH factors	NLP-extracted, aOR (95% CI)*	Structured, aOR (95% CI)*	Combined, aOR (95% CI)*
Social problems, Financial problems	2.39 (2.22, 2.59)	2.48 (2.18, 2.82)	2.34 (2.17, 2.52)
Social problems, Housing instability	2.47 (2.28, 2.68)	2.41 (2.11, 2.74)	2.38 (2.20, 2.57)
Social problems, Legal problems	3.01 (2.70, 3.36)	2.60 (2.33, 2.92)	2.84 (2.60, 3.09)
Social problems, Violence	2.94 (2.70, 3.21)	3.37 (2.82, 4.02)	2.69 (2.49, 2.91)
Social problems, Barriers to care	2.46 (2.27, 2.67)	-	2.41 (2.23, 2.61)
Social problems, Transition of care	2.15 (2.02, 2.29)	-	2.12 (2.00, 2.26)
Social problems, Food insecurity	2.19 (1.87, 2.56)	-	2.15 (1.85, 2.50)
Social problems, Non-specific psychosocial needs	-	2.15 (1.96, 2.36)	2.21 (2.04, 2.40)
Financial problems, Housing instability	2.45 (2.25, 2.66)	2.10 (1.86, 2.35)	2.35 (2.17, 2.55)
Financial problems, Legal problems	3.00 (2.68, 3.35)	3.16 (2.66, 3.75)	2.98 (2.70, 3.27)
Financial problems, Violence	2.83 (2.58, 3.11)	3.54 (2.87, 4.36)	2.69 (2.47, 2.94)
Financial problems, Barriers to care	2.35 (2.16, 2.57)	-	2.33 (2.14, 2.54)

© 2023 Mitra A et al. *JAMA Network Open.*

Financial problems, Transition of care	2.07 (1.94, 2.22)	-	2.08 (1.94, 2.22)
Financial problems, Food insecurity	2.19 (1.87, 2.56)	-	2.20 (1.88, 2.57)
Financial problems, Non-specific psychosocial needs	-	2.45 (2.14, 2.80)	2.37 (2.16, 2.60)
Housing instability, Legal problems	3.16 (2.81,3.56)	2.91 (2.45, 3.45)	3.14 (2.84, 3.47)
Housing instability, Violence	2.95 (2.67, 3.27)	3.13 (2.52, 3.88)	2.77 (2.52, 3.04)
Housing instability, Barriers to care	2.42 (2.20, 2.66)	-	2.41 (2.20, 2.64)
Housing instability, Transition of care	2.08 (1.93, 2.23)	-	2.08 (1.93, 2.23)
Housing instability, Food insecurity	2.21 (1.87, 2.61)	-	2.18 (1.85, 2.56)
Housing instability, Non-specific psychosocial needs	-	2.64 (2.30, 3.03)	2.46 (2.23, 2.72)
Legal problems, Violence	3.44 (3.03, 3.89)	3.31 (2.72, 4.02)	3.34 (3.01, 3.70)
Legal problems, Barriers to care	3.00 (2.65, 3.39)	-	2.93 (2.63, 3.25)
Legal problems, Transition of care	2.78 (2.51, 3.07)	-	2.77 (2.54, 3.02)
Legal problems, Food insecurity	2.73 (2.20, 3.39)	-	2.66 (2.20, 3.23)

© 2023 Mitra A et al. *JAMA Network Open.*

Legal problems, Non-specific psychosocial needs	-	2.65 (2.39, 2.94)	2.74 (2.48, 3.01)
Violence, Barriers to care	2.82 (2.55, 3.12)	-	2.65 (2.41, 2.91)
Violence, Transition of care	2.51 (2.32, 2.71)	-	2.31 (2.15, 2.48)
Violence, Food insecurity	2.54 (2.09, 3.09)	-	2.39 (1.99, 2.88)
Violence, Non-specific psychosocial needs	-	2.93 (2.50, 3.43)	2.83 (2.55, 3.15)
Barriers to care, Transition of care	2.00 (1.86, 2.14)	-	1.99 (1.86, 2.13)
Barriers to care, Food insecurity	2.19 (1.86, 2.59)	-	2.20 (1.87, 2.60)
Barriers to care, Non-specific psychosocial needs	-	-	2.40 (2.16, 2.66)
Transition of care, Food insecurity	1.95 (1.70, 2.24)	-	1.96 (1.70, 2.25)
Transition of care, Non-specific psychosocial needs	-	-	2.23 (2.05, 2.42)
Food insecurity, Non-specific psychosocial needs	-	-	2.48 (2.04, 3.02)

*Each model was adjusted for socio-demographic variables, psychiatric symptoms, substance abuse, pain, patient disability, clinical comorbidities and all SDOH in its group.

eTable 10. Associations of SDOH with Veterans' death by suicide

© 2023 Mitra A et al. *JAMA Network Open.*

eReferences

1. Assessment and Management of Patients at Risk for Suicide (2019) - VA/DoD Clinical Practice Guidelines. https://www.healthquality.va.gov/guidelines/MH/srb/. Accessed June 25, 2022.
2. Liu Y, Ott M, Goyal N, et al. RoBERTa: A Robustly Optimized BERT Pretraining Approach. July 2019. http://arxiv.org/abs/1907.11692. Accessed January 17, 2020.
3. Lee J, Yoon W, Kim S, et al. BioBERT: a pre-trained biomedical language representation model for biomedical text mining. *Bioinformatics*. September 2019. doi:10.1093/bioinformatics/btz682
4. Alsentzer E, Murphy J, Boag W, et al. Publicly Available Clinical BERT Embeddings. In: Association for Computational Linguistics (ACL); 2019:72-78. doi:10.18653/v1/w19-1909
5. Li F, Jin Y, Liu W, Rawat BPS, Cai P, Yu H. Fine-tuning bidirectional encoder representations from transformers (BERT)–based models on large-scale electronic health record notes: An empirical study. *J Med Internet Res*. 2019;21(9):e14830. doi:10.2196/14830
