# Supplementary material for: Associations Between Natural Language Processing–Enriched Social Determinants of Health and Suicide Death Among US Veterans
Source: JAMA Netw Open. 2023 Mar 15;6(3):e233079. doi: 10.1001/jamanetworkopen.2023.3079 (PMC10018322; doi:10.1001/jamanetworkopen.2023.3079)
Supplement: Supplement 2. — Data Sharing Statement [file jamanetwopen-e233079-s002.pdf]

Data Sharing Statement

Mitra. Associations Between Natural Language Processing-Enriched Social Determinants of Health and Suicide Death Among US Veterans. *JAMA Netw Open*. Published March 15, 2023. doi:10.1001/jamanetworkopen.2023.3079

Data
Data available: No
